# Waiting times for radiotherapy: variation over time and between cancer networks in southeast England

**DOI:** 10.1038/sj.bjc.6602463

**Published:** 2005-03-22

**Authors:** D Robinson, T Massey, E Davies, R H Jack, A Sehgal, H Møller

**Affiliations:** 1 Thames Cancer Registry, Division of Cancer Studies, Guy's King's and St Thomas’ School of Medicine, Capital House, 42 Weston Street, London SE1 3QD, UK

**Keywords:** radiotherapy, waiting times, cancer networks, trends

## Abstract

The aim of this study was to investigate variations in the length of time that patients with cancer wait from diagnosis to treatment with radiotherapy. A total of 57 426 men and 71 018 women diagnosed with cancer between 1992 and 2001 and receiving radiotherapy within 6 months of diagnosis were identified from the Thames Cancer Registry database. In total, 12 sites were identified for which a substantial number or proportion of patients received radiotherapy: head and neck, oesophagus, colon, rectum, lung, nonmelanoma skin cancer, breast, uterus, prostate, bladder, brain and non-Hodgkin's lymphoma. Median waiting times from diagnosis to radiotherapy were calculated, together with the proportion of patients who received radiotherapy within 60 days of diagnosis, and analysed by year of diagnosis, cancer site, deprivation quintile, age at diagnosis, sex and cancer network of either residence or treatment. Logistic regression was used to adjust the proportion receiving treatment within 60 days for the effects of the other factors. There were significant differences in the proportions receiving radiotherapy within 60 days between different networks and different cancer sites, which remained after adjustment. Median waiting times varied from 42 to 65 days across networks of residence, with the adjusted proportion treated within 60 days ranging from 44 to 71%. There was no difference between male and female patients after adjustment for the other factors, particularly site. There was a highly significant trend over time: the median wait increased from 45 days in 1992 to 76 days in 2001, while the adjusted proportion being treated within 60 days declined by almost a half, from 64 to 35%, over the same period.

Over the past 5 years the UK government has introduced a series of policies designed to decrease the time that patients wait from the first suspicion of cancer to its investigation and definitive treatment. In 1999 a policy proposed in 1997, stipulating a maximum 2-week wait between urgent referral by a general practitioner and first hospital appointment, was implemented for patients with suspected breast cancer (Department of Health, 1997). This policy was subsequently extended to include all cancer sites. Additional targets relating to the investigation, diagnosis and treatment of cancer have since been defined (Department of Health, 2000, 2004). In particular, a target of a maximum 2-month wait from urgent GP referral to treatment for all cancers has been set for 2005.

A number of UK studies have focused attention on waiting times for cancer patients. A large retrospective survey of all English patients diagnosed in October 1997 (Spurgeon *et al*, 2000) found that the waiting time from initial referral to outpatient appointment and to treatment varied according to tumour type, whether the initial referral was coded urgent, and by the region where treatment was undertaken. For nonurgent referrals, patients with breast cancer experienced the shortest waits (median wait of 14 days to first outpatient appointment and 35 days to first definitive treatment) while patients with prostate cancer waited the longest (median 41 days to first outpatient appointment and 111 days to first definitive treatment). Patients with lung cancer experienced intermediate waits (median 12 days to first outpatient appointment and 47 days to first definitive treatment).

A number of smaller studies from single centres have also raised concern. A study of 75 head and neck cancer patients in Liverpool (Jones *et al*, 2002) reported a mean wait from GP to specialist consultation of 5.1 weeks. The longest delay was the time taken to the start of primary radiotherapy, which ranged from 4 to 18 weeks (mean 10.3 weeks). Patients waited twice as long for radiotherapy than for surgery as the definitive treatment. An audit of 29 lung cancer patients awaiting radical radiotherapy in Glasgow (O’Rourke and Edwards, 2000) found a median delay between the first hospital visit and starting radiotherapy of 94 days. During this time, six potentially curable patients became incurable.

The majority of studies investigating waiting times for cancer patients have focused on whether longer waits affect survival. A systematic review by Richards *et al* (1999) of 87 studies of breast cancer found that patients with delays of 3 months or more from the onset of symptoms to treatment had 12% lower survival than those with shorter delays. However, longer delays were associated with more advanced stage at time of treatment, and were not associated with reduced survival once the effect of stage was taken into account.

A recent study by Mikeljevic *et al* (2004) found an increased relative risk of death in breast cancer patients treated with conservative surgery who subsequently waited longer than 9 weeks for radiotherapy. Likewise, there is some evidence that a longer wait for postoperative radiotherapy adversely affects survival for patients with head and neck cancers, small-cell-lung cancer and high-grade gliomas (Seel and Foroudi, 2002).

Conversely, a study of early glottic laryngeal carcinoma (Brouha *et al*, 2000) found that the waiting time between diagnosis and radiotherapy (median 43 days, range 9–180 days) had no effect on the outcome. Similarly, the time to treatment did not affect survival in patients with non-small-cell lung cancer in a UK study (Bozcuk and Martin, 2001), or in those with pharyngeal cancer in a study in Finland (Koivunen *et al*, 2001).

The interaction between delay and survival is complex. A study of endometrial cancer in Scotland (Crawford *et al*, 2002) found that women who experienced the longest delays to surgery were more likely to survive, suggesting that general practitioners were communicating information to consultants, enabling them to respond faster to women at higher risk and decreasing their delay between referral and first hospital visit. A similar interpretation emerged from a study of breast cancer patients in Denmark (Afzelius *et al*, 1994), where the prognosis was better for patients with a long doctor's delay compared with those with a short delay. The authors concluded that doctors are effective at distinguishing between more and less aggressive tumours.

A recent study of breast cancer patients in Germany (Arndt *et al*, 2003) found a U-shaped association between treatment delay and stage at diagnosis, with the highest proportions of late-stage tumours in women with either very short or very long delays, thus suggesting that two different mechanisms are at work. Women presenting with advanced disease may be diagnosed and treated more quickly, because of the clearer clinical picture, while in those presenting with very early symptoms diagnosis may be delayed by false negative findings at the initial examination, resulting in further advanced disease at the time of treatment.

Regardless of survival, any delay in the pathway from definitive cancer diagnosis to treatment is likely to increase anxiety and should be minimised if possible. In a previous study (Robinson *et al*, 2003), in which we investigated the immediate effect of the introduction of the government ‘2-week wait’ target, we found that the wait from first hospital appointment to first treatment for women with breast cancer in southeast England was highly dependent on the type of treatment. The small group of women who received radiotherapy as their first treatment experienced long delays, with little more than half being treated within the target time of 5 weeks from first hospital appointment. The present study extends this analysis to cancers other than breast cancer, and to a longer period of time. We have examined the delay between the diagnosis of cancer at a number of sites and the commencement of radiotherapy treatment, and the variation in waiting times across cancer networks and over calendar time.

## SUBJECTS AND METHODS

The Thames Cancer Registry (TCR) covers a population of around 14 million people in London and the southeast of England. All 608 952 registered cases of cancer that were diagnosed between 1992 and 2001 and resident within the TCR catchment area were examined. Patients were regarded as having received radiotherapy if a valid date of radiotherapy treatment within 6 months of diagnosis was recorded. The date of diagnosis is defined as the date on which cancer was confirmed by the best of the diagnostic tests performed (ideally histological or cytological confirmation) or, if not available, the date of admission to hospital for the malignancy or, if not admitted, the date of first outpatient consultation. The date of radiotherapy is the date on which active radiotherapy treatment of any kind was begun.

**Figure 1:**
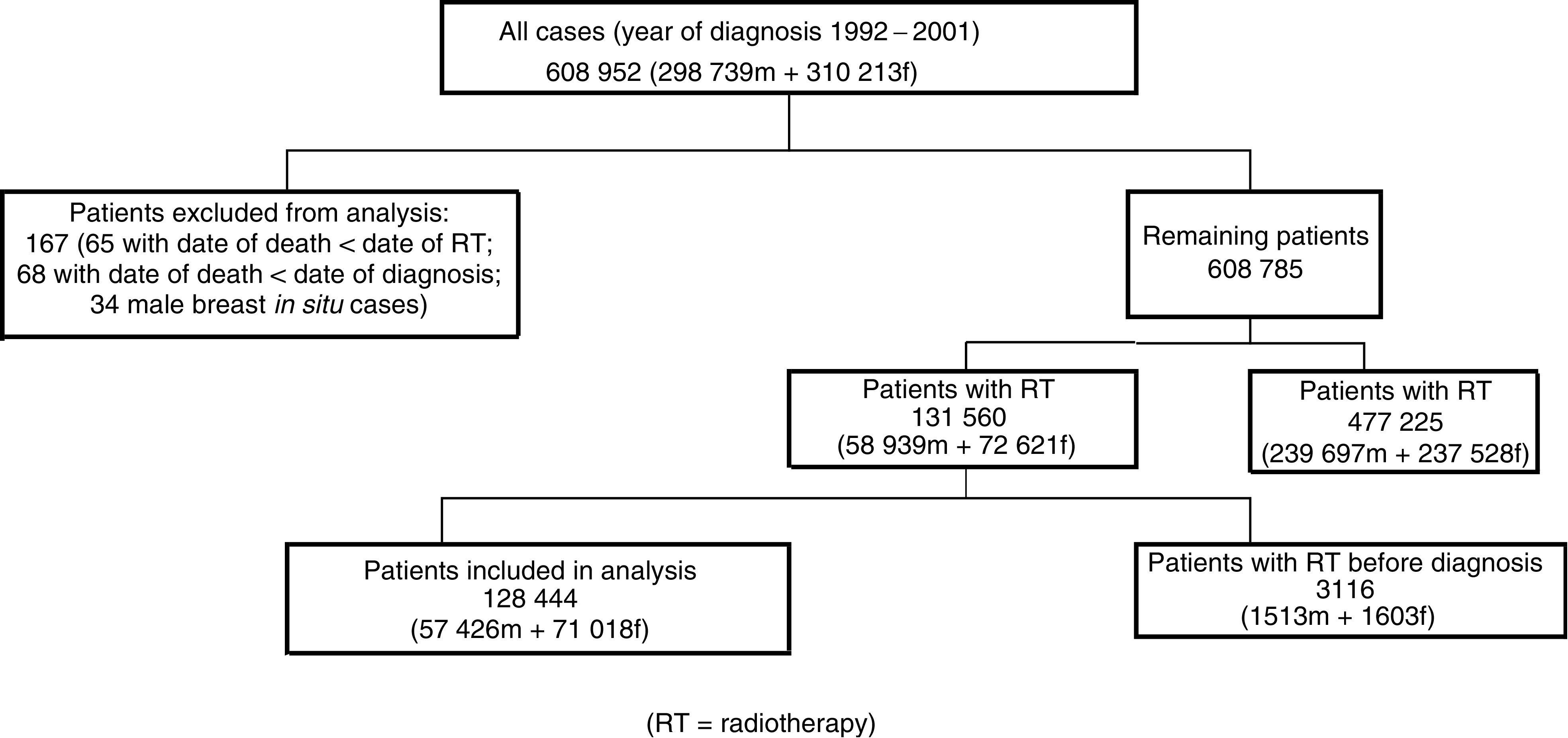
Flow chart of patient selection.

Figure 1 shows how cases were selected for the study. In total, 133 patients, recorded as dying before they received either their diagnosis or radiotherapy treatment, were excluded, together with 34 cases of male breast cancer *in situ*. Invasive male breast cancers (575 cases) were retained. Overall, 131 560 patients had received radiotherapy. A small proportion (3116 cases, 2.4%) who were recorded as receiving radiotherapy prior to their diagnosis were subsequently excluded, and the analysis based on the 128 444 patients who received radiotherapy after diagnosis.

In total, 12 cancer sites were identified for which a substantial number or proportion of cases had received radiotherapy. These were cancers of the head and neck, oesophagus, colon, rectum, lung, female breast, uterus, prostate, bladder and brain, nonmelanoma skin cancer, and non-Hodgkin's lymphoma. Female breast cancers were subsequently subdivided into invasive and *in situ* cases, and brain tumours (which included tumours of the meninges and of the pituitary and pineal glands) into malignant and benign cancers. The ‘benign’ group also contained tumours of ‘uncertain or unknown behaviour’. Two thirds of the cases in this group who received radiotherapy had pituitary tumours. The ‘bladder’ site included both invasive and *in situ* cases; all other specified sites comprised only invasive cases. Finally, all remaining cases were combined into an ‘other’ group. For the male patients, this included the invasive breast cancer cases.

Age at diagnosis was calculated and grouped into five bands. Cancer network of residence was determined from the postcode of the patient's address at the time of diagnosis, and network of treatment from the hospital at which radiotherapy was provided. Socioeconomic deprivation was assessed on the basis of postcode of residence. Each subject was assigned to an appropriate ward, and hence to a quintile of the ward income domain score, a subcomponent of the indices of multiple deprivation (IMD) 2000 for England (Department of the Environment, Transport and the Regions, 2000).

Logistic regression analysis was performed, with the dependent variable being the receipt of radiotherapy within 60 days of diagnosis. This provided a convenient cutoff close to the overall median waiting time of 56 days, and corresponds to the 2-month target for urgent referrals set for 2005 by the NHS Cancer Plan. Factors included in the model were: year of diagnosis, site, IMD quintile, age group, sex, and network of either residence or treatment. Adjustment for length of survival after diagnosis was achieved by including a dichotomous variable indicating whether the subject had survived for less than 6 months or longer. This simple adjustment gave similar results to an analysis that excluded patients who died within 6 months of diagnosis, but enabled us to retain more cases for analysis.

An alternative analysis was performed by fitting a Cox proportional hazards model to the data. The results, which are not presented here, were qualitatively the same as those from the logistic model. We chose to present the latter as they are easier to interpret. We present both the crude and adjusted proportions receiving radiotherapy within 60 days, to give an indication of the degree of sensitivity to case-mix adjustment. For each factor, the proportions were adjusted to allow for differences in all of the other factors, along with length of survival as detailed above.

## RESULTS

Table 1 shows the number of different cancers diagnosed during the study period, together with the number and percentage of cases receiving radiotherapy. There were a number of striking differences between the sexes: for head and neck cancer and for brain tumours, the proportion of women receiving radiotherapy was substantially lower than the corresponding proportion in men.

The results of the logistic regression analysis are shown in Table 2. There was a highly significant variation in the proportion of cases receiving radiotherapy within 60 days of diagnosis between cancer network of residence. This remained after adjustment for the other factors. The median wait for radiotherapy varied from 42 to 65 days. When network of treatment was included in the model instead of network of residence, the pattern was almost identical (results not shown).

**Figure 2:**
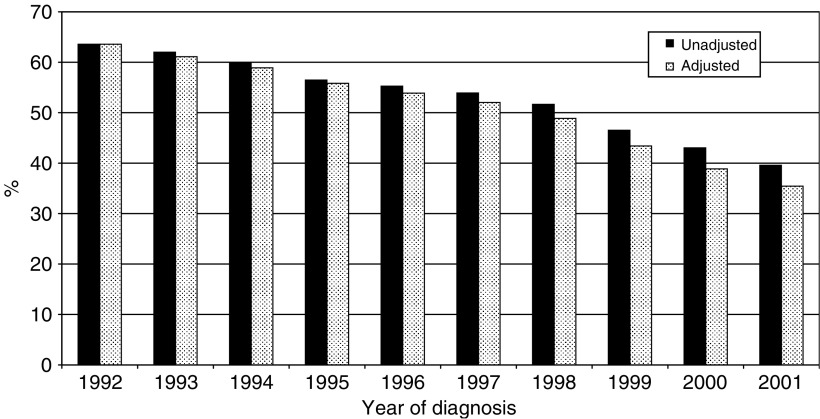
Proportion of patients receiving radiotherapy within 60 days, by year of diagnosis.

There was a highly significant trend in radiotherapy waiting times over time. The median wait increased from 45 days in 1992 to 76 days in 2001, while the proportion being treated within 60 days declined from 64 to 40% over the same period. In the adjusted model the gradient became even steeper, falling from 64 to 35%. This is shown graphically in Figure 2.

There was significant variation between cancer sites: the longest waits (and smallest percentages treated within 60 days) were seen for cancers of the colon, breast, prostate and bladder, while the shortest waits (and largest percentages treated within 60 days) were found in brain and lung cancer cases. The differences between different cancers were not sensitive to adjustment for the other factors.

**Figure 3:**
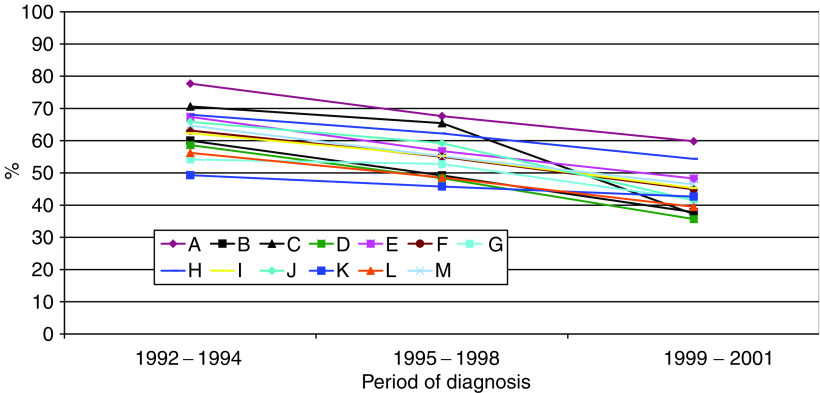
Unadjusted proportion of patients receiving radiotherapy within 60 days, by cancer network of residence and period of diagnosis.

**Figure 4:**
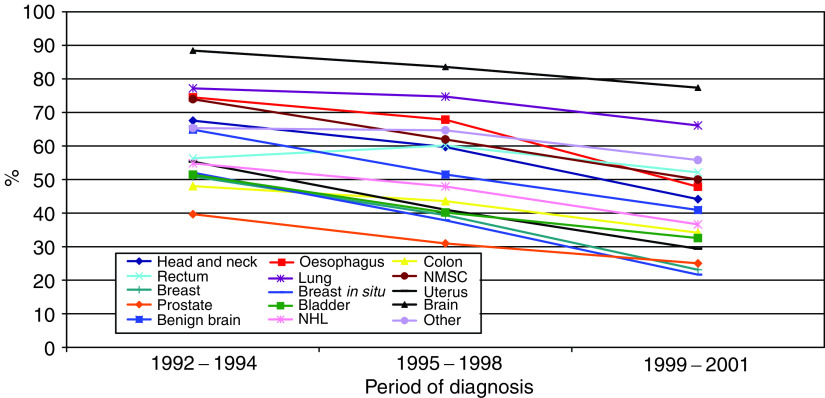
Unadjusted proportion of patients receiving radiotherapy within 60 days, by cancer site and period of diagnosis.

Figure 3 shows the unadjusted proportion of cases in each cancer network of residence who received radiotherapy within 60 days, by different time periods. The first period (1992–1994) represents the pre-Calman/Hine era, and the later period (1999–2001) follows the introduction of government targets on waiting times. Figure 4 shows a similar plot of unadjusted proportions by cancer site. These graphs demonstrate the largely consistent differences between cancer networks and between different types of cancer, and the highly consistent downward trend over time.

Before adjustment for the other factors, there was a significant trend in the proportion being treated within 60 days across the IMD quintiles, with a greater proportion in the most deprived group (quintile 5). This gradient disappeared completely after adjustment for case-mix, and in the adjusted estimates there is a tendency for slightly shorter waiting times in the most prosperous group.

There was also a significant trend related to age, with a greater proportion of the older cases being treated within 60 days. This trend was attenuated, but remained statistically significant, after adjustment for the other factors.

Before adjustment, there was a highly significant difference between the sexes, with 58% of men and 49% of women receiving radiotherapy within 60 days. However, after adjustment for the other factors this difference disappeared completely.

## DISCUSSION

The main findings of this study are the differences in waiting times for radiotherapy between different cancer networks and different cancer sites, and the large increase in waiting times observed over the 10-year period of the study. Even after adjustment for other factors, large variation remained between different cancer networks, with the proportion receiving radiotherapy within 60 days of diagnosis ranging from 44 to 71%.

The difference in the proportion receiving radiotherapy within 60 days between male and female patients was completely negated after adjustment for the other factors, particularly site. A large proportion of women with breast cancer (38%) or uterine cancer (41%) received radiotherapy – sites for which the waiting time was relatively high. Conversely, a smaller proportion of women than men received radiotherapy for head and neck and brain cancers, for which waiting times were shorter.

In the context of large variation in radiotherapy waiting times between different cancers, it was not surprising to find variation between socioeconomic groups, simply because the incidence of different cancers varies between such groups. The tendency, after case-mix adjustment, towards a slightly shorter waiting time in the most prosperous group may be due to preferential treatment of this group, but the difference is not large (52 *vs* 50–51% treated within 60 days) and only marginally statistically significant, despite the very large number of cases.

Although these findings may appear dramatic, our study has a number of shortcomings. Our measure of waiting time (from diagnosis to radiotherapy) is a crude one, containing a number of intervals where delay is possible. There is a wait after diagnosis for referral for radiotherapy, followed by a planning stage, and then a further wait for treatment. From the point of view of the radiotherapy department, a more relevant waiting time to analyse would be from referral for radiotherapy to treatment, but our data set did not have the dates required to calculate this. In any case, what is most important to the individual patient is the total waiting time for treatment.

**Figure 5:**
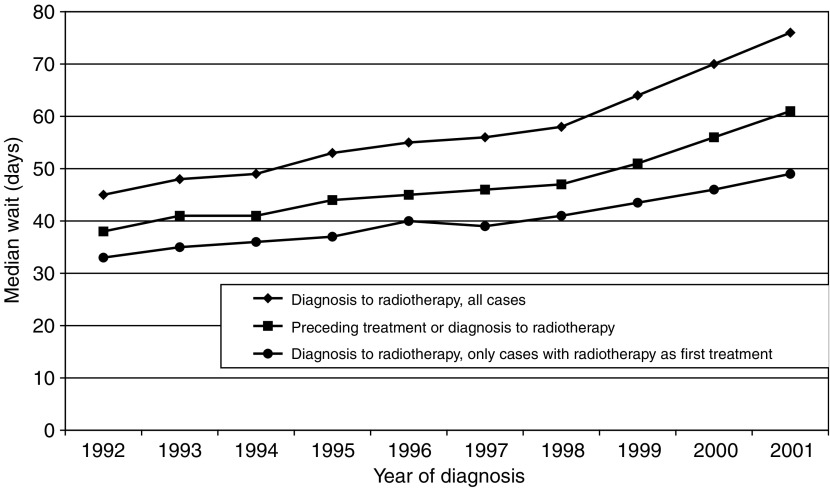
Median waiting times by year of diagnosis.

We have not allowed for the effect on waiting times of concomitant therapy such as surgery or chemotherapy. Again, this was because we wanted to explore the broader picture. However, repeating the analysis using a ‘truncated’ waiting time, defined as the wait to radiotherapy from the immediately preceding treatment in those patients who received some other form of therapy between diagnosis and radiotherapy, or as the wait from diagnosis to radiotherapy for those patients with no intermediate treatment, produced qualitatively similar results. Figure 5 shows the trends over time in the median values of these two waits, along with the median wait from diagnosis to radiotherapy in the smaller group of patients for whom radiotherapy was the first treatment. Although the ‘truncated’ wait and the wait from diagnosis in those receiving radiotherapy as their first treatment are shorter, the patterns are similar for the three measures.

There is also a possibility that radiotherapy treatment has been under-ascertained in our data set (i.e. radiotherapy details are not recorded), although this would be unlikely to be related in any systematic way to the factors we have investigated.

A recent Canadian study (Johnston *et al*, 2004) of waiting times from diagnosis to radiotherapy in patients with breast, lung, colorectal or prostate cancer found differences in median waiting times between the different cancer sites similar to those seen in our study. Some of the observed differences between cancer sites can be explained by the nature of the tumours concerned or the associated radiotherapy. For example, head and neck cancers tend to proliferate quickly and hence rapid treatment is required. Likewise, radiotherapy for lung cancer is often largely palliative and hence treatment can be delivered quickly and may involve only a few fractions of radiation. The observed differences between males and females in the proportions receiving radiotherapy for certain types of cancer (Table 1) may be due to the varying frequency of different subtypes of cancer between men and women.

We found that older patients had shorter waiting times for radiotherapy. Older people tend to receive a less radical treatment regime (e.g. no concomitant chemotherapy) and hence are likely to proceed to radiotherapy earlier. In the Canadian study (Johnston *et al*, 2004), waiting times decreased with age for breast and prostate cancer, but not for colorectal or lung cancer.

Our finding of increasing waiting times over the period of the study is consistent with previous reports. Mikeljevic *et al* (2004) found that the mean interval between conservative breast surgery and radiotherapy increased from 5 to 12 weeks over a 10-year period from the late 1980s to the late 1990s. Factors likely to contribute to this effect include availability of trained staff and equipment and increases in workload due to either an increased incidence of cancer or changes in the management of tumours over time, with larger numbers being referred for radiotherapy. The latter would appear to be ruled out in our study: Table 2 shows that the total numbers of registered cancers and also the numbers and proportions of patients referred for radiotherapy have been relatively stable over the study period. However, only radiotherapy within 6 months of diagnosis was recorded on our database. It is possible that there may be an increased number of cases receiving radiotherapy more than 6 months after diagnosis in the more recent years. If this is so, and if these had been included in our analysis, then the observed trends over time would be even more dramatic.

Another factor contributing to the lengthening waiting times could be the increase in treatment complexity associated with the recent widespread applications of 3-D conformal and intensity-modulated radiotherapy. We were not able to explore the possible effect on waiting times of changes in the number of patients receiving palliative radiotherapy on recurrence.

The trend of increasing waiting times appears to have accelerated since 1999 (see Table 2 and Figure 4), when the government targets on waiting times from GP referral to first hospital appointment were introduced. In an earlier study (Robinson *et al*, 2003), we showed that this had a knockon effect, with decreased waiting times during this earlier part of the cancer pathway being offset by increased waits from first hospital appointment to treatment – particularly for radiotherapy.

Although our data only permit analysis up to 2001, a recent report from the Royal College of Radiologists (2003) found that the proportion of patients waiting longer than 4 weeks to start radical radiotherapy had increased from 28% in 1998 to 81% in 2002. They concluded that ‘waiting lists are longer than ever’, that ‘modern treatment is inhibited widely by lack of up to date equipment’, and that ‘many departments cannot function at their full capacity because they do not have the staff to allow them to do so’.

Likewise, a national audit of 2498 patients seen during 1 week in 2003 at all 55 radiotherapy centres in the UK (Ash *et al*, 2004) found that median waiting times had increased by 2 weeks since 1998 for both radical and adjuvant radiotherapy, and that fewer patients were being treated within the standards for good practice set by the Joint Collegiate Council for Oncology (JCCO). The percentage of patients waiting longer than the maximum times advised by the JCCO had increased from 32% in 1998 to 72% in 2003 for radical treatments, from 25 to 60% for palliative treatments, and from 39 to 62% for adjuvant treatments.

Waiting time is only a single aspect of care, and the overall quality of care should be assessed with consideration of other dimensions as well. Lilford *et al* (2004) have argued vigorously against the use of comparative outcome measures (such as league tables and star ratings) in a health care setting. Short waits are not necessarily equivalent to best clinical practice. We are currently analysing waiting times for a number of specific cancer sites separately and in greater detail, taking into account the effects of additional treatment and the extent of disease at diagnosis, and also investigating the effects of longer waits on survival.

## Figures and Tables

**Table 1:** Numbers and percentages of cases receiving radiotherapy by sex and cancer site. All years combined (1992–2001)

	**Males**		**Females**	
**Cancer site**	**Total cases**	**No. (%) with radiotherapy**	**Total cases**	**No. (%) with radiotherapy**
Head and neck	10 617	6398 (60.3%)	6686	3290 (49.2%)
Oesophagus	8738	1872 (21.4%)	5699	1468 (25.8%)
Colon	20 821	667 (3.2%)	23 329	555 (2.4%)
Rectum	14 229	3433 (24.1%)	11 478	2398 (20.9%)
Lung	51 621	17 103 (33.1%)	30 534	9286 (30.4%)
Nonmelanoma skin cancer	10 834	1676 (15.5%)	6884	954 (13.9%)
Breast	—	—	89 164	34 162 (38.3%)
Breast *in situ*	—	—	5955	1192 (20.0%)
Uterus	—	—	17 181	7051 (41.0%)
Prostate	55 047	9211 (16.7%)	—	—
Bladder	22 757	3453 (15.2%)	8788	1367 (15.6%)
Brain	5498	2363 (43.0%)	4374	1582 (36.2%)
Brain, benign	2720	316 (11.6%)	3725	232 (6.2%)
Non-Hodgkin's lymphoma	10 618	1595 (15.0%)	9338	1477 (15.8%)
Other	83 623	9339 (11.2%)	85 411	6004 (7.0%)
				
Total	297 123	57 426 (19.3%)	308 546	71 018 (23.0%)

**Table 2:** Median waiting times, and crude and adjusted proportions of patients treated with radiotherapy (RT) within 60 days from date of diagnosis, by various factors

	**Total cases**	**No. (%) of cases receiving RT**	**Median wait (days)**	**Propn. treated within 60 days (%)**	**Adjusted^a^ propn. treated within 60 days (%)**
*Cancer network of residence*					
A^b^	12 611	1442 (11.4%)	42	67.3	70.6
B	72 783	16775 (23.0%)	62	49.1	46.4
C^b^	28 771	5547 (19.3%)	52	56.9	58.8
D^b^	42 015	10 001 (23.8%)	63	48.1	46.8
E	58 188	11 707 (20.1%)	50	58.1	54.9
F	45 889	9947 (21.7%)	54	55.0	53.4
G	59 750	12 094 (20.2%)	61	49.9	47.1
H	33 032	7327 (22.2%)	49	61.3	61.4
I^c^	54 469	10 809 (19.8%)	56	53.7	53.7
J	58 050	13 231 (22.8%)	54	56.0	55.0
K	60 907	11 003 (18.1%)	65	45.7	43.7
L^b^	14 386	2652 (18.4%)	62	48.9	45.7
M	64 226	15 818 (24.6%)	52	55.8	55.4
Other/not known	592	91 (15.4%)	49	64.8	58.9
Test for heterogeneity				*χ*^2^=1079.7 (*P*<0.001)	*χ*^2^=1263.6 (*P*<0.001)
					
*Year of diagnosis*					
1992^c^	59 518	12 181 (20.5%)	45	63.6	63.6
1993	56 926	11 654 (20.5%)	48	62.0	61.1
1994	59 066	12 855 (21.8%)	49	59.8	58.9
1995	59 746	12 982 (21.7%)	53	56.5	55.8
1996	62 526	13 787 (22.0%)	55	55.3	53.9
1997	62 912	14 411 (22.9%)	56	53.9	52.0
1998	61 827	13 717 (22.2%)	58	51.7	48.9
1999	61 773	13 221 (21.4%)	64	46.5	43.4
2000	62 241	12 297 (19.8%)	70	43.0	38.9
2001	59 134	11 339 (19.2%)	76	39.6	35.4
Test for trend				*χ*^2^=2675.6 (*P*<0.001)	*χ*^2^=3107.9 (*P*<0.001)
					
*Cancer site*					
Head and neck	17 303	9688 (56.0%)	54	57.4	56.3
Oesophagus	14 437	3340 (23.1%)	46	63.8	53.0
Colon	44 150	1222 (2.8%)	70	42.5	36.1
Rectum^c^	25 707	5831 (22.7%)	54	56.5	56.5
Lung	82 155	26 389 (32.1%)	35	73.1	61.9
Nonmelanoma skin cancer	17 718	2630 (14.8%)	48	63.1	57.2
Breast	89 164	34 162 (38.3%)	71	38.3	38.9
Breast *in situ*	5955	1192 (20.0%)	74	35.0	37.7
Uterus	17 181	7051 (41.0%)	68	42.3	40.9
Prostate	55 047	9211 (16.7%)	95	31.0	29.5
Bladder	31 545	4820 (15.3%)	69	42.2	32.2
Brain	9872	3945 (40.0%)	35	82.9	82.0
Brain, benign	6445	548 (8.5%)	56	53.7	55.0
Non-Hodgkin's lymphoma	19 956	3072 (15.4%)	64	46.7	42.0
Other	169 034	15 343 (9.1%)	45	62.3	56.1
Test for heterogeneity				*χ*^2^=11381.8 (*P*<0.001)	*χ*^2^=5899.7 (*P*<0.001)
					
*IMD quintile*					
1^c^	112 942	25 112 (22.2%)	58	52.0	52.0
2	119 629	25 400 (21.2%)	58	51.9	50.5
3	120 101	25 681 (21.4%)	57	52.7	50.5
4	128 505	26 811 (20.9%)	55	53.9	50.3
5	123 740	25 350 (20.5%)	54	55.6	50.7
Not known	752	90 (12.0%)	50	63.3	65.6
Test for trend				*χ*^2^=85.3 (*P*<0.001)	*χ*^2^=4.78 (*P*=0.03)
					
*Age at diagnosis*					
<50^c^	70 609	20 341 (28.8%)	63	47.6	47.6
50–59	79 565	24 294 (30.5%)	62	48.8	50.4
60–69	132 666	34 725 (26.2%)	58	51.5	50.3
70–79	180 470	35 024 (19.4%)	52	56.4	53.1
80+	142 359	14 060 (9.9%)	43	65.4	60.6
Test for trend				*χ*^2^=1258.8 (*P*<0.001)	*χ*^2^=349.9 (*P*<0.001)
*Sex*					
Male^c^	297 123	57 426 (19.3%)	50	58.4	58.4
Female	308 546	71 018 (23.0%)	62	49.1	58.4
Test for heterogeneity				*χ*^2^=1097.6 (*P*<0.001)	*χ*^2^=0.0 (*P*=0.95)
					
All cases	605 669	128 444 (21.2%)	56	53.2	

a Adjusted for all other factors, and for survival.

b TCR covers only a part of this network.

c Reference group.

Tests for heterogeneity and trend exclude ‘other’ and ‘not known’ categories.
